# Call for an Evidence-Based Consensus on Outcome Reporting in Tinnitus Intervention Studies

**DOI:** 10.3389/fmed.2017.00042

**Published:** 2017-04-21

**Authors:** Alain Londero, Deborah A. Hall

**Affiliations:** ^1^Service ORL et CCF, Hôpital Européen G. Pompidou, Paris, France; ^2^NIHR Nottingham Biomedical Research Centre, Nottingham, UK; ^3^Otology and Hearing Group, Division of Clinical Neuroscience, School of Medicine, University of Nottingham, Nottingham, UK

**Keywords:** tinnitus, Delphi survey, trial reporting, core outcome set, patient-reported complaints

Tinnitus is a very common symptom affecting 5.1–42.7% of the population (1) and is frequently seen in family medicine and primary care settings. Despite its considerable socioeconomic relevance (2), real progress in developing an effective cure for tinnitus has been fruitless (3). In most of the cases, the proposed therapies remain palliative; aiming at alleviating the negative consequences of tinnitus. Although the need for effective management options for tinnitus is clear, methodological and reporting quality of clinical trials have been low (4, 5) making useful recommendations and practical guidelines for family medicine and primary health-care practitioners almost impossible to draw. Indeed, Baguley and colleagues (6) concluded that, with the exception of cognitive behavior therapy for tinnitus, evidence for the effectiveness of different treatment strategies is insufficient (pp. 1605).

The CONSORT statement^1^ is perhaps the most well-known guideline for solving problems arising from inadequate reporting of randomized controlled trials, but other tinnitus-specific statements have been around since the 1990s. Unfortunately, these recommendations have not yet transformed standards in the tinnitus field so far. Indeed, recent systematic reviews of published clinical trials aiming at evaluating tinnitus therapeutic interventions have shown that reporting is still flawed by poor methodology and poor reporting (4, 5).

In this opinion, we discuss the selection and reporting of outcomes; perhaps, the most important aspect of determining whether a treatment works for patients and whether this treatment should be implemented in the medical practice either in primary or secondary settings. Selecting an appropriate outcome for determining clinical efficacy is one of those key trial design decisions. As Noble eloquently put it: “critical to any form of treatment for tinnitus is the reliance placed on measures to assess the effectiveness of the intervention” (pp. 20) (7). Just over 10 years later, Landgrebe et al. (8) made the same point stressing that “assessment of outcome is probably the single most important factor in conducting a clinical trial in tinnitus” (pp. 9). This is so because in clinical trials, therapeutic benefit is evaluated according to its effect on primary (and secondary) outcome measures that should be purposefully chosen according to the complaints (domains) of tinnitus considered to be most important from the perspective of determining therapeutic benefit (9). More specifically, the primary outcome measure is that which confirms whether or not the primary hypothesis is supported by the data. It is typically a variable relating to clinical efficacy but could also be one relating to safety, tolerability, or quality of life, if that is the primary research question. Generally speaking, the primary outcome should also be the endpoint that is clinically relevant from the patients’ perspective and to health-care providers’ in their everyday practice, not just significant from a statistical point of view. In support of this, the ICH E9 states that “The primary variable should be that variable capable of providing the most clinically relevant and convincing evidence directly related to the primary objective of the trial” (pp. 5) (10).

Bearing in mind that tinnitus is a subjective condition for which patients experience a diversity of complaints, there is no straightforward outcome instrument. Outcome reporting typically relies on self-report, often in the form of a multi-item tinnitus questionnaire that asks questions about a range of complaints (not all of which are the same across existing questionnaires). A number of important issues have been raised and debated over the years, but many of those concerns remain unresolved. Table 1 summarizes conclusions/recommendations about clinical trial outcomes in tinnitus. Although this may not be exhaustive, it nevertheless serves to illustrate the status of the field spanning across three decades.

**Table 1:** **Concluding remarks or recommendations about clinical trial outcomes in tinnitus made in various review articles**.

Reference	Conclusions and recommendations concerning outcomes
Tyler (29)	Benefit should be measured with established questionnaires and with measures of the magnitude of tinnitus. A persuasive tinnitus treatment will be one that shows a large treatment effect, can be generalized across patients and clinicians, is specific and credible, and changes the way we think about tinnitus
Tyler et al. (9)	Several scaling procedures are available, but we believe a 100-point interval scale is superior. Several validated and reliable questionnaires are available and can serve as adequate primary measures. Secondary measures that quantify the magnitude of the tinnitus should also be obtained
Langguth et al. (15)	It was generally agreed that a questionnaire is required that is specifically designed for the assessment of treatment outcomes, and which is validated in many languages and in many cultural and socioeconomic groups. The consensus agreement is that at the present time one validated questionnaire, which can be Tinnitus Handicap Inventory (THI), Tinnitus Handicap Questionnaire (THQ), TRQ, or Tinnitus Questionnaire (TQ), is an essential part of patient assessment. Therapeutic trials should use one of these questionnaires also as outcome measurement. Assessment of tinnitus severity with at least one additional questionnaire is highly recommended
Meikle et al. (11)	While the tinnitus questionnaires that are currently available provide valuable information on which to base diagnostic and screening decisions, they were not originally developed in such a way as to maximize their sensitivity to treatment-related changes in tinnitus. As a result, their construct validity for measuring treatment benefit has not received appropriate attention
Tyler et al. (9)	When the treatment is intended to reduce the tinnitus, we recommend measuring the magnitude of the tinnitus. We provide arguments and data to support the use of the THQ as a measure of the reaction to the tinnitus. We suggest that the current quality of life measures are not valid for measuring lifestyle effects of alleviating tinnitus. A clinically meaningful effect should represent a valid and reliable statistical change for an individual
Meikle et al. (13)	It is to be hoped that investigators will address the need for information about the responsiveness of all the various types of tinnitus measures. The fact that measures of sensory impairment versus functional disability and handicap each provide unique insights into treatment-related changes in tinnitus reinforces the notion that both approaches are needed for insightful assessment of tinnitus treatment outcomes
Hesser (30)	If we restrict the assessment to one particular aspect of tinnitus-related disability, definitive claims about overall treatment benefits will be difficult to make. Moreover, the measures we use need to be validated and psychometrically robust. Although several psychometrically examined measures are available to assess tinnitus impact and severity (e.g., THI, Tinnitus Reaction Questionnaire), there is no standard outcome measure that is obligatory to include in a trial. I do believe that treatment evaluations within the field would not only benefit from calculating and reporting average effects but also must rely on data on the individual level, e.g., as clinical significant change, in determining the effects of treatment
Kamalski et al. (12)	The Health-Related-Quality of Life (HR-QoL) instruments used in tinnitus trials (THI, TQ, TRQ, TSI, THQ, and TSQ) appear not to be validated to measure effectiveness of interventions. Using tests or instruments that are valid and reliable is a crucial component of research quality, and both should therefore be studied before final conclusions can be drawn from the questionnaires in upcoming clinical trials. The validity, reliability, and responsiveness of each tinnitus-specific HR-QoL should be studied before final conclusions can be drawn regarding the utility of these questionnaires in future clinical studies
Landgrebe et al. (8)	Basic requirements for clinical trials in tinnitus include: – Definition of one or more main outcome measure(s) (i.e., a validated tinnitus questionnaire). – THI should be included in every trial at least as secondary outcome to improve inter-study comparability
Newman et al. (31)	Although psychometrically robust measures of tinnitus HR-QoL do exist, there is no unanimity in, for example, what tests should be included in the tinnitus assessment, and how studies of HR-QoL should be conducted. The current authors suggest that future studies employ more rigorous designs and contain (minimally) the following characteristics: (1) utilization of randomized control groups and blinding; (2) appropriate statistical testing including “dropouts” that should be used in an “intention to treat” analysis rather than elimination from the final data set; (3) long-term follow-up assessment to evaluate responsiveness; (4) appropriate inclusion criteria to avoid “ceiling” and “floor” effects; and (5) suitable sample sizes based on the application of power analyses
Fackrell et al. (14)	– We recommend that the “gold standard” would be to carry out a systematic review of the literature before selecting any given tinnitus questionnaire for a service audit or clinical trial. – In addition to the measurement properties, selection might also give consideration to the suitability of the tinnitus questionnaire for the study population, the potential burden of completing the questionnaire (e.g., length, question difficulty, emotional impact of certain questions), and the practical aspects (e.g., copyright costs, complexity of scoring method)
Hall et al. (27)	The overall ambition of the working group is to establish an international standard for outcome measurements in clinical trials of tinnitus. The standard will be achieved by a two-step effort to produce core outcome sets of domains and instruments that harmonize viewpoints across both professional and patient stakeholder groups. A roadmap has been proposed, which sets out a provisional plan for delivery. This roadmap reflects the two-step process with Stage 1 identifying and agreeing on outcome domains and Stage 2 identifying and agreeing on outcome instruments
Plein et al. (4)	(There is) a need in the literature for high-quality tinnitus research that is adequately randomized, ensures adequate follow-up, and does not exclude a large range of common otologic conditions that can result in tinnitus, which would result in improved external validity. Analysis of external validity is essential to the development of further guidelines and should be taken into account if we hope to develop recommendations that are of most benefit to clinicians and patients
Hall et al. (5)	– Generic names and terms such as “handicap” and “severity” perpetuate the difficulty that many trialists experience in understanding what construct(s) a particular questionnaire instrument measures. – Safety, tolerability, side effects, and withdrawals might be domains that all inform the measurement of adverse events. To improve trial reporting, we draw attention to the specialized CONSORT guidelines for reporting harms-related issues in a randomized controlled trial. – We advise caution if pooling findings from the THI in a meta-analysis since it is unclear whether all translations achieve equivalence with the British original

Our observations are as follows:

(i) Are instruments really validated for use in assessing tinnitus treatment-related change?

While early conclusions implied that questionnaires are the “best” primary outcomes and that adequate “validated” questionnaires exist for this purpose, by 2007 some researchers were beginning to challenge the validity of existing tinnitus questionnaires for use in assessing treatment-related change (11). In 2010, a particularly critical evaluation of the psychometric properties of six of the commonly used multi-item questionnaires assessing tinnitus burden, including the Tinnitus Handicap Inventory, TQ, and Tinnitus Handicap Questionnaire, was published by Kamalski et al. (12). For each identified tinnitus-specific Health-Related-Quality of Life questionnaire, they systematically searched for published details regarding the questionnaire’s test characteristics including number of domains, construct validity, internal consistency, reproducibility, and responsiveness. Like Meikle et al. (13), they were critical that none of the six questionnaires assessed had been validated for evaluative purposes, which is necessary to be useful in clinical trials. In particular, responsiveness, which measures the ability to detect a clinically important change over time, had not been reported for any of the six instruments.

Fackrell et al. (14) raised a new issue about the dynamic nature of psychometric properties. What questionnaire properties hold for one patient population might not for another. Indeed, for this reason we prefer the term psychometric “exploration” not “validation,” and we hope that questionnaire developers might be sympathetic to adaptations in order to maintain equivalence across cultures [see also Ref. (15)].

(ii) Can we reduce the diversity of outcome instruments?

Two recent systematic reviews of outcome instruments in tinnitus trials have confirmed unacceptable heterogeneity in measurement tools (4, 5) For example, we found 78 different primary outcome instruments across 228 trials (5). This makes comparisons between studies elusive.

(iii) What is a clinically meaningful effect?

Tyler et al. (9) highlighted the importance of benefit from the individual patient experience, although this psychometric property of tinnitus questionnaires has generally not been investigated or quantified [see Meikle et al. (16), for a good example].

In the race to develop and utilize tinnitus questionnaires in our research, we are losing sight of understanding “what” it is that needs to be measured at the expense of “how” it is measured. Do we really know which tinnitus-related complaints are the most relevant both from the first and second line health-care providers’ and patients’ perspectives? We would argue not. Primary care physicians and patients in particular have been left out of the questionnaire development process.

Involving primary care physicians and patients in developing outcome reporting standards would go a long way to resolving heterogeneity in outcome assessment and ensuring its relevance. It could then help the tinnitus community to focus efforts on conducting an appropriate psychometric exploration of whatever are the preferred instruments. As an important first step, we are therefore leading a consensus exercise to develop an agreed minimum reporting set of outcomes for trials of interventions in tinnitus. What is urgently needed are targeted discussions around “what” needs to be measured for each therapeutic approach (sound therapies, pharmacological therapies, psychological interventions, and neuromodulation) since not all interventions seek to alleviate the same tinnitus-related complaints.

Such consensus for a minimal (core) set of outcome measurements that should be used in every clinical trial clearly calls for a predefined, multidisciplinary (including patients and primary care professionals), international, and methodologically driven clinimetric approach (17). The rationale underpinning this scientific approach of outcome assessment has already been theorized. For example, the Core Outcome Measures in Effectiveness Trials (COMET) initiative brings together from all over the world researchers interested in the development and application of agreed standardized “Core Outcome Sets” (COS) (18). A COS represents the minimum that should be measured and reported in all clinical trials for a specific condition (19). COS could also be suitable for a use in clinical audit or research other than randomized trials. One should note that COS are not limitative. If necessary, other outcomes might be added to those in the relevant COS in a particular trial. But, it is recommended that all the COS items should be collected and reported, making it easier for the results to be compared, contrasted, and merged as appropriate. Indeed, similar international initiatives aiming at harmonizing outcome assessment are already existing such as for eczema (HOME for Harmonizing Outcome Measures for Eczema) (20) or, in the auditory field, for hearing loss (ICF for International Classification of Functioning, disability, and health core sets for hearing loss) (21). Then, we urge the tinnitus community to adopt and adapt these international standards of outcome definition and evaluation to the tinnitus field.

Deciding which outcome domains should be in this minimal core set requires a great deal of interactions and collaboration between stakeholders, professionals, and patients. This objective lends itself well to an international and multidisciplinary effort. We propose to follow a Delphi methodology to reach this goal. Delphi might be defined as “a method for structuring a group communication process so that the process is effective in allowing a group of individuals, as a whole, to deal with a complex problem” (22). Delphi methodology has been already proposed with success to reach a consensus in a variety of complex medical issues [e.g., see Ref. (23–25)]. Delphi methodology has also been used in the tinnitus field (26). Adapting a Delphi protocol to define a consensus core set of domains and a core set of instruments for tinnitus assessment in clinical trials is the aim of the recently launched Core Outcome Measures in Tinnitus (COMiT) initiative on behalf of the EU COST BM1306 action.^2^ The activities of our COMiT initiative are registered on the website of the COMET initiative^3^ according to roadmap that plans out a program of work. The first step is aimed at identifying and agreeing on core domains (27). It has started with two systematic reviews in order to establish existing knowledge and practice: the first review reflects the view of professional stakeholders by systematically looking at the current reported outcome domains in tinnitus intervention studies. The results of this review have already been published according to the PRISMA checklist of items to include when reporting a systematic review (5) establishing which outcome domains and outcome instruments have been measured in recent registered and published clinical trials. The second one represents the opinion of tinnitus people who experience the condition and/or their significant others by systematically searching in the literature and the internet the dimensions or domains that relate to tinnitus perceived intrusiveness. This second study will summarize the findings of narrative syntheses of qualitative data to establish which domains are important to patient and their significant others (28).

The data synthesis arising from both of these reviews will inform our online Delphi process, which will seek a consensus about what outcome domains are important both from health-care professionals’ and patients’ perspectives. The methods for reaching consensus will use an iterative series of questionnaires, with an international multidisciplinary panel of patients, clinicians, and other professional stakeholder groups (such as industry) all contributing with their views. We plan to conduct three independent Delphi processes devoted to different types of interventions (sound-, psychology-, and pharmacology-based interventions) that may require different COS. This Delphi process is illustrated in Figure 1. It is expected that the whole Delphi process will cover the 4-year period of the COST Action BM1306 grant. Our strong belief is that this innovative effort will improve not only trial reporting in the tinnitus domain but also the practical guidelines recommended in primary or secondary care settings for this common condition mistakenly considered to be always untreatable.

**Figure 1:**
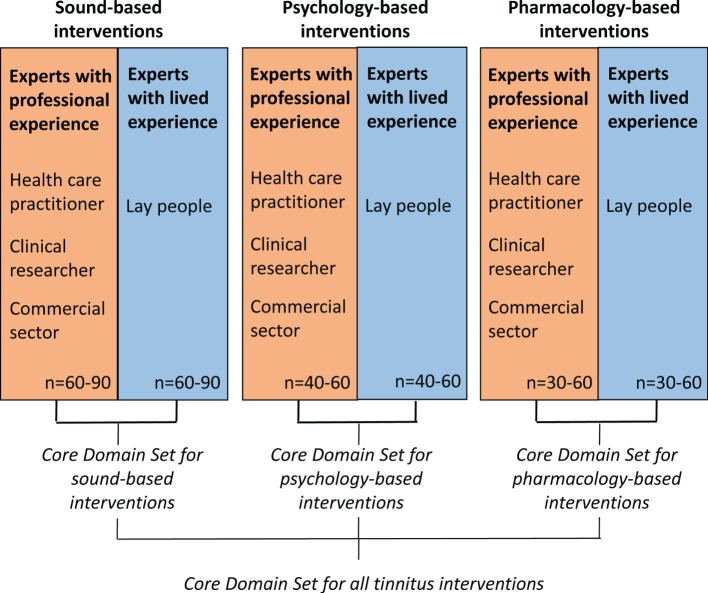
**Proposed Delphi survey strategy to identify different core outcome sets that are appropriately tailored according to different tinnitus interventions**.

## Author Contributions

AL and DAH participated in conceiving, drafting, and revising the article and gave final approval of the version to be submitted.

## Conflict of Interest Statement

The authors declare that the research was conducted in the absence of any commercial or financial relationships that could be construed as a potential conflict of interest.

## Footnotes

^1^www.consort-statement.org/.

^2^http://www.cost.eu/COST_Actions/bmbs/BM1306.

^3^www.comet-initiative.org.
